# Supplementary material for: Improved Assembly of Metagenome-Assembled Genomes and Viruses in Tibetan Saline Lake Sediment by HiFi Metagenomic Sequencing
Source: Microbiol Spectr. 2022 Dec 8;11(1):e03328-22. doi: 10.1128/spectrum.03328-22 (PMC9927493; doi:10.1128/spectrum.03328-22)

Supporting information

Improved assembly of MAGs and viruses in Tibetan saline lake sediment by HiFi metagenomic sequencing

Ye Tao[1,2], Fan Xun[1,2], Cheng Zhao[1,2], Zhendu Mao[1,2], Biao Li[1], Peng Xing[1*], and Qinglong L. Wu[1,2,3]

[1]State Key Laboratory of Lake Science and Environment, Nanjing Institute of Geography and Limnology, Chinese Academy of Sciences, Nanjing 210008, China

[2]University of Chinese Academy of Sciences, Beijing 100049, China

[3]Center for Evolution and Conservation Biology, Southern Marine Sciences and Engineering Guangdong Laboratory (Guangzhou), Guangzhou 511458, China

*Author for correspondence, Prof. Dr. Peng Xing, Email: pxing@niglas.ac.cn; Phone: 86 25 86882112; Fax: 86 25 5771 4759

Running head: HiFi-read sequencing for extremophiles

Supplementary Tables

Table S1 Physiochemical information of the sediment samples from five sites within Lake Cuochuolong.

**Site**	**CCL1**	**CCL2**	**CCL3**	**CCL4**	**CCL5**
Longitude(ºE)	85.4063	85.40487	85.40274	85.40061	85.39687
Latitude(ºN)	29.12109	29.12113	29.122	29.12309	29.12333
Altitude(m)	4610	4616	4616	4619	4623
Temp(°C)	14.4	14.8	14.6	14.9	15.3
pH	9.3	9.24	9.27	9.26	9.25
Conductivity(μS/cm)	34297	34782	34574	34773	35293
Salinity (‰)	27.35	27.78	27.59	27.77	28.23
sediment TOC (%)	2.69	0.67	0.85	1.22	1.10
sediment TN (%)	0.21	0.04	0.05	0.09	0.05
LOI (g/g dw sediment)	0.22	0.23	0.24	0.22	0.19

Table S2 The number of 16S/18S genes obtained by eight assemblers.

	HiFi			Hybrid			NGS	
Assembler	HiCanu	metaFlye	HiFiasm_meta	MaSuRCA	HybridmetaSPAdes	Operams	Megahit	metaSPAdes
16S rRNA	69	154	384	318	233	354	263	222
Partial 16S rRNA	17	42	113	70	193	277	223	200
Complete 16S rRNA	52	112	271	248	40	77	40	22
Complete 16S-OTUs	23	76	108	130	35	73	40	33
18S rRNA	81	150	385	318	115	176	126	93
Partial 18S rRNA	80	141	367	315	111	167	126	93
Complete 18S rRNA	1	9	18	3	4	9	0	0
Complete 18S-OTUs	1	7	7	2	4	8	0	0

Table S3 Summary of total MAGs identified in this study. The MAG quality classification: 'High' (≥90% completeness, ≤5% contamination), 'Medium' (≥50% completeness, ≤10% contamination), and others were 'Low'. 'QS' means the quality score for MAG (completeness – 5 × contamination). 'High/Medium' MAGs were annotated by GTDB-Tk.

The information was saved in the file Table_S3.xlsx.

删除了: mean

Table S4 Statistical summary of the two cMAGs and their closest genomes.

	ccl-hifiasmmeta.153	GCA_020164535.1	ccl-flye.84	GCF_003387225.1	GCF_004135935.1
Genome Length (bp)	3,024,023	3,036,757	3,028,805	3,292,724	3,298,588
N50 (bp)	2,560,458	458,041	3,028,805	931,588	3,298,588
Contig Number	2	15	1	8	1
GC%	36.6	36.6	62.5	62.2	62.3
Gene Number	2,808	2,849	3,174	3,525	3,391
Gene Density (genes/kb)	0.928	0.938	1.047	1.07	1.028
tRNA	38	37	46	44	45
16S rRNA copy	3	1	1	1	1

Table S5 Summary of viral genomes and the corresponding vOTUs.

The information was saved in the file Table_S5.xlsx.

Table S6 The genome length and family information of complete viral genomes.

Methods	ID	Length (bp)	Family
metaFlye	contig_2047	49167	Siphoviridae
metaFlye	contig_3808	115239	Myoviridae
metaFlye	contig_4001	33878	Siphoviridae
metaFlye	contig_4396	139208	Myoviridae
metaFlye	contig_4507	99275	Podoviridae
metaFlye	contig_4599	42086	Podoviridae
metaFlye	contig_4601	34327	Podoviridae
metaFlye	contig_4617	81194	Podoviridae
metaFlye	contig_4636	38795	Siphoviridae
metaFlye	contig_4674	66614	Siphoviridae
metaFlye	contig_4777	24417	Siphoviridae
metaFlye	contig_4825	105314	Podoviridae
metaFlye	contig_4868	38481	Podoviridae
metaFlye	contig_4923	139613	Myoviridae
metaFlye	contig_4947	331546	Myoviridae
metaFlye	contig_4969	38946	Siphoviridae
metaFlye	contig_5055	29706	Siphoviridae
metaFlye	contig_533	40074	Myoviridae
metaFlye	contig_820	80705	Siphoviridae
HiFiasm_meta	s1426.ctg001639c	40221	Podoviridae
HiFiasm_meta	s1601.ctg001836c	47105	Siphoviridae
HiFiasm_meta	s2495.ctg002855c	43729	Siphoviridae
HiFiasm_meta	s2912.ctg003323c	39241	Siphoviridae
HiFiasm_meta	s3034.ctg003459c	65692	Podoviridae
HiFiasm_meta	s3209.ctg003658c	34119	Siphoviridae
HiFiasm_meta	s4718.ctg005371c	28460	Podoviridae
HiFiasm_meta	s5458.ctg006209c	37672	Podoviridae
HiFiasm_meta	s557.ctg000633c	131003	Siphoviridae
HiFiasm_meta	s7064.ctg008018c	39664	Podoviridae

Supplementary Figures

Figure S1 Location of Lake Cuochuolong on the Tibetan Plateau and the sampling sites within the lake.

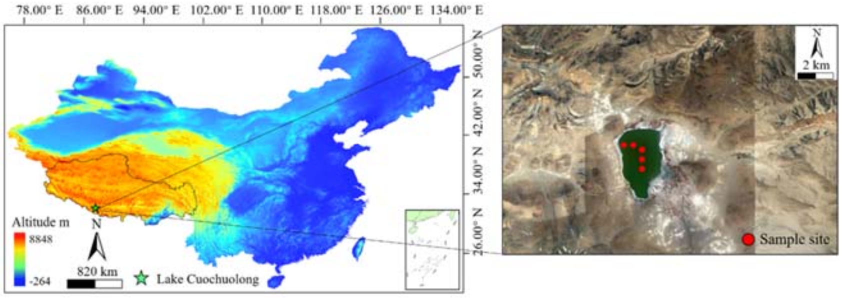

Figure S2 Krona chart of detected species at different taxonomic levels using HiFi (A) and NGS (B) reads by Kraken2, respectively.

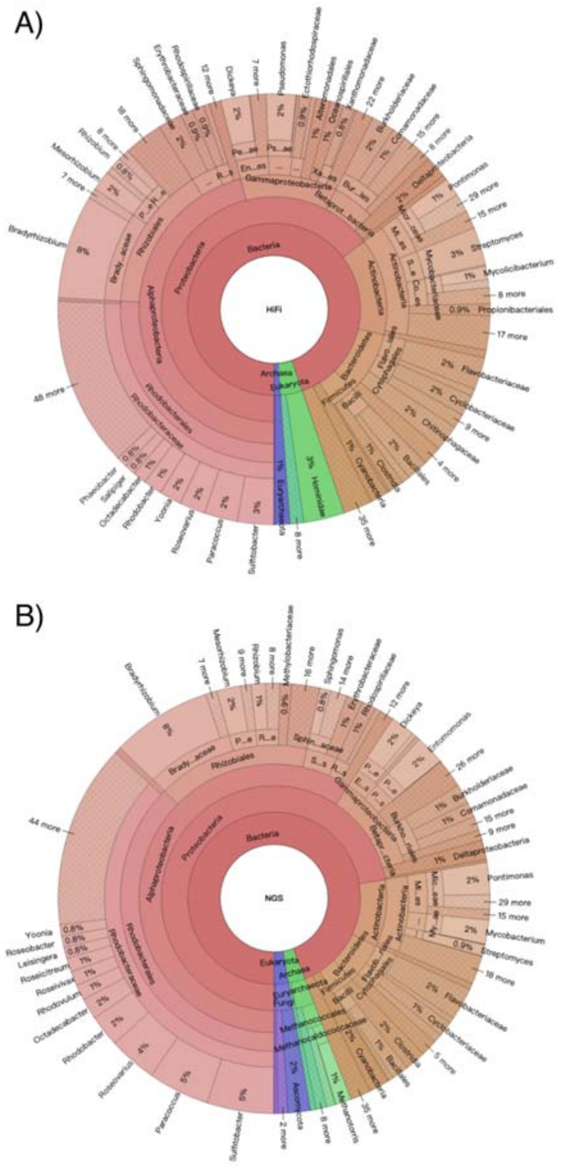

Figure S3 Taxonomic affiliations of community composition between HiFi and NGS reads at the phylum level.

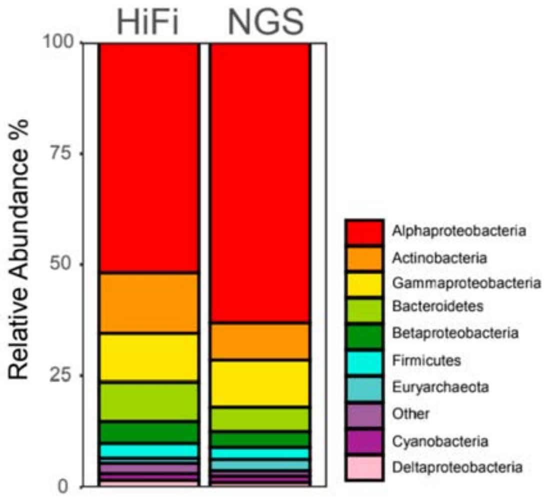

Figure S4 Phylogenomic tree and heatmap based on ANI values of cMAG ccl−hifiasmmeta.15 and its close genomes.

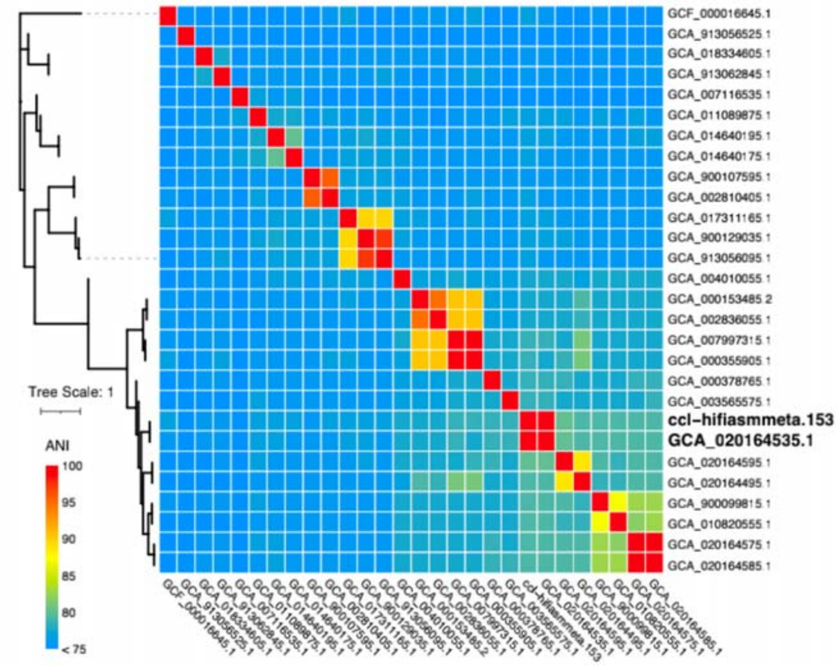

Figure S5 Two cMAGs circle maps. (A) ccl−hifiasmmeta.153 had two circular chromosomes. (B) ccl−flye.84. The outermost circle of the circle diagram is the identification of the genome size, the second circle, and the third circle are the CDSs on the forward and reverse strands, and different colors indicate the functional classification of the coding genes. The fourth circle is the GC content, and the fifth circle is the G+C skew.

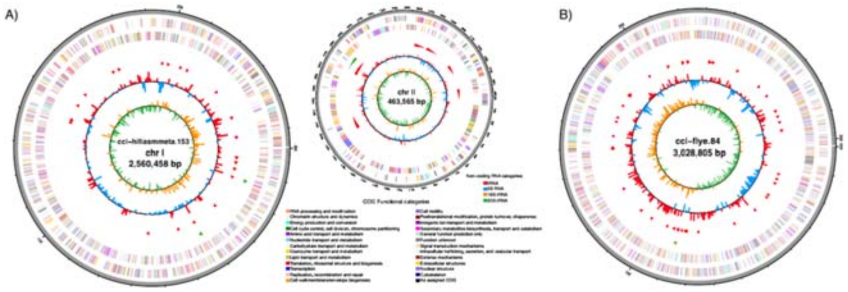

Figure S6 Proteomic trees based on 1-S_G distance matrix of 29 cvOTUs and closest known viruses via ViPTree (https://www.genome.jp/viptree). Genera and subfamilies are delineated with colored squares and colored circles, respectively. The red star indicates the cvOTUs.

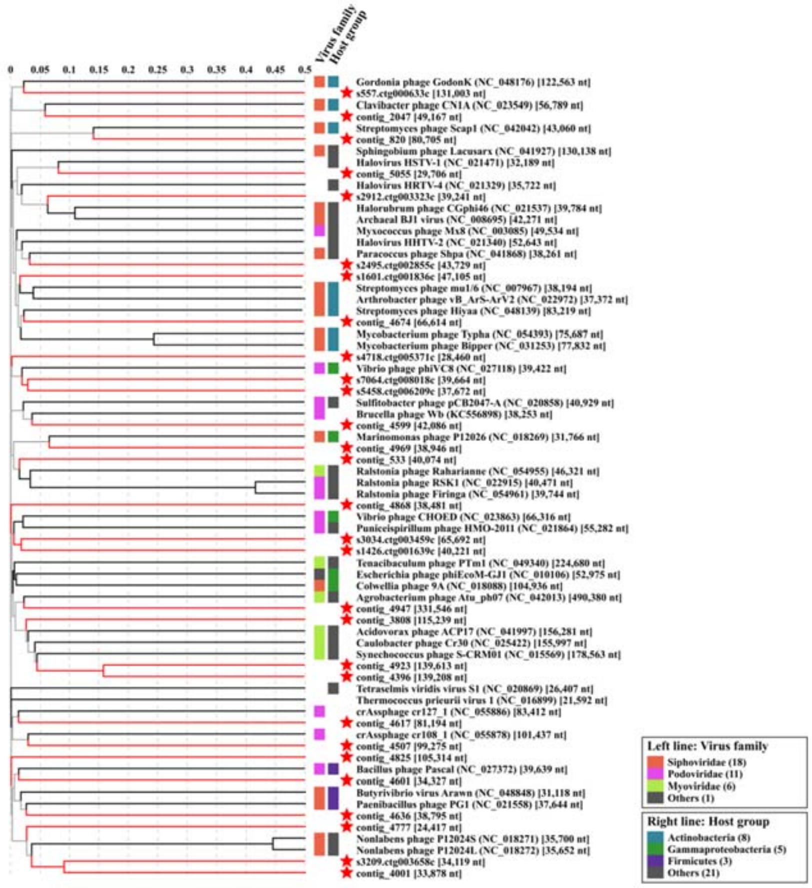

Figure S7 Heatmap of auxiliary metabolic genes detected in cvOTUs and genome circle maps for two cvOTUs containing *phnP* gene.

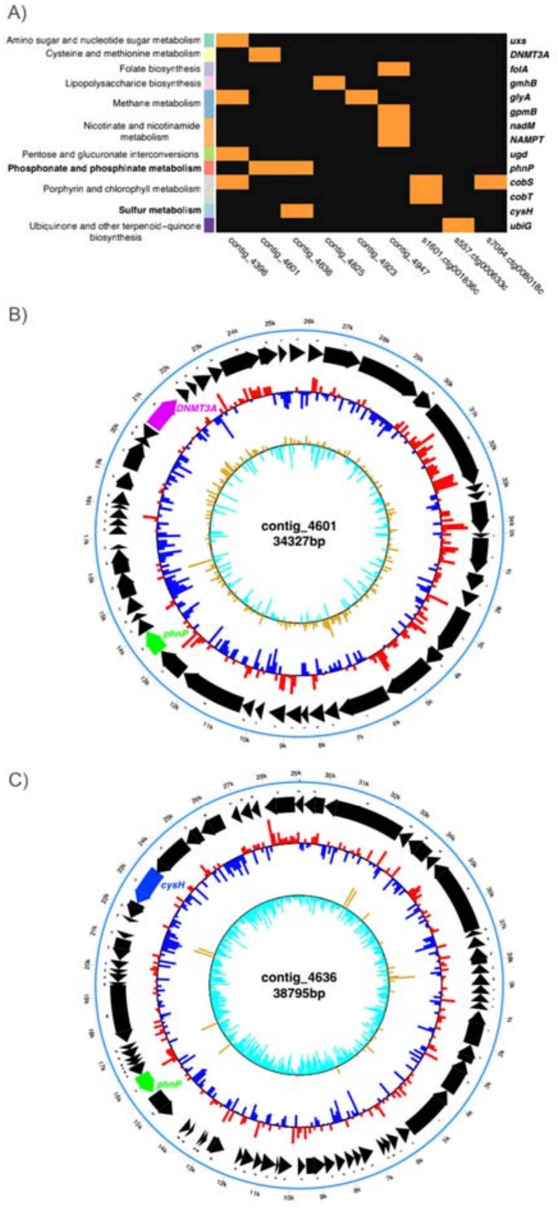

Figure S8 BGCs analysis. (A) The number of novel and known BGCs from eight assemblies. The distribution of different classes of complete (B) and partial (C) BGCs from eight assemblies.

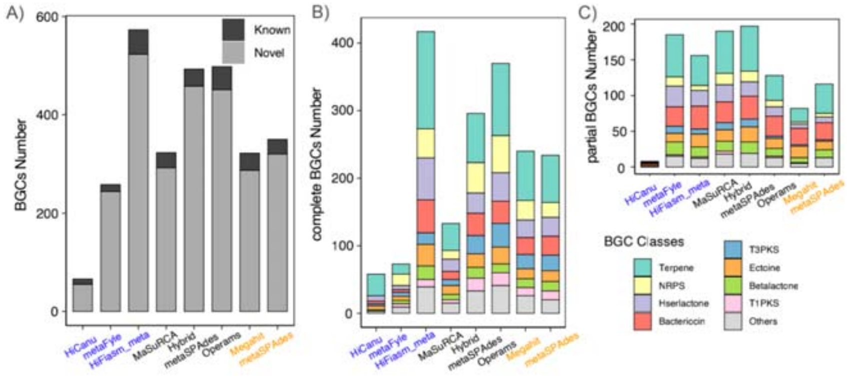

Figure S9 Estimated coverage of the metagenomic data sets obtained in this study. (A) Fitted Nonpareil curve and estimated coverage. The maximum coverage was 0.94. (B) The genome size of Megahit assembles under increasing NGS data.

删除了: and the

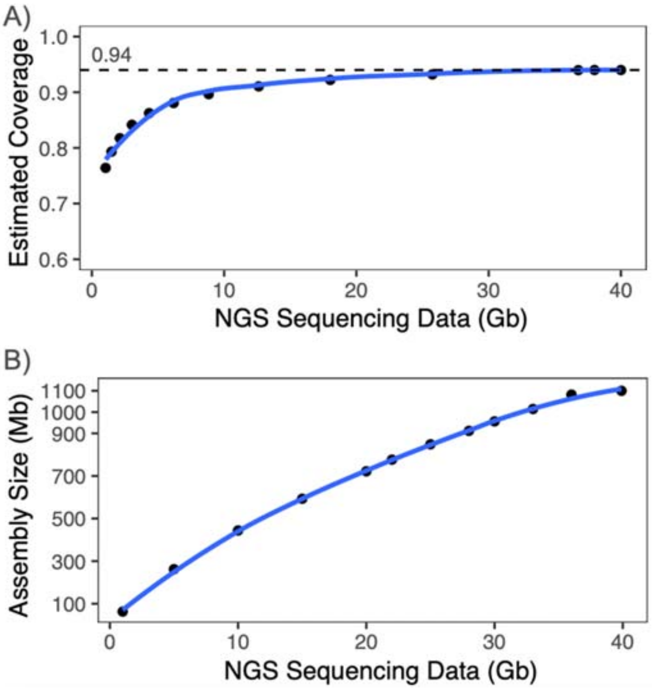

Supplement: Supplemental file 1 — Tables S1, S2, S4, and S6 and Fig. S1 to S9. Download spectrum.03328-22-s0001.pdf, PDF file, 0.6 MB [file spectrum.03328-22-s0001.pdf]
